# Herd management and subsistence practices as inferred from isotopic analysis of animals and plants at Bronze Age Politiko-*Troullia*, Cyprus

**DOI:** 10.1371/journal.pone.0275757

**Published:** 2022-10-26

**Authors:** Suzanne E. Pilaar Birch, Mary Metzger, Elizabeth Ridder, Steven Porson, Steven E. Falconer, Patricia L. Fall

**Affiliations:** 1 Department of Anthropology and Department of Geography, University of Georgia, Athens, Georgia, United States of America; 2 School of Instructor Education, Vancouver Community College, Vancouver, British Columbia, Canada; 3 Department of Liberal Studies, California State University San Marcos, San Marcos, California, United States of America; 4 Department of Geography, University of North Carolina, Charlotte, Charlotte, North Carolina, United States of America; 5 Department of Anthropology, University of North Carolina, Charlotte, Charlotte, North Carolina, United States of America; University of Padova: Universita degli Studi di Padova, ITALY

## Abstract

The Bronze Age village of Politiko-*Troullia*, located in the foothills of the copper-bearing Troodos mountains of central Cyprus, was occupied ~2050–1850 cal BCE. Excavated evidence shows that community activities included copper metallurgy (ore processing, smelting and casting), crop cultivation, and rearing of livestock. Faunal analysis reveals day-to-day subsistence practices that included consumption of sheep, goat, cattle, and pig, as well as community-scale ritual feasting focused on fallow deer, *Dama dama mesopotamica*. In this paper, we present bone collagen stable isotope data from these taxa to infer how these animals were managed. We incorporate stable isotope baselines calculated from modern cereal grains and compare these to archaeological seeds from Politiko-*Troullia*. Mean values of δ^13^C and δ^15^N cluster for livestock consistent with a diet of C3 plants, with a wider range in goats that suggests free-browsing herds. Higher δ^15^N values in cattle may reflect supplemental feeding or grazing in manured fields. Plant isotope values suggest livestock diets were predominantly composed of cultivated taxa. In contrast, deer and pig bones produce more negative mean δ^13^C and δ^15^N values suggesting that the villagers of Politiko-*Troullia* complemented their management of domesticated animals with hunting of wild deer and feral pigs in the woodlands surrounding their village.

## Introduction

The human settlement of Cyprus in the early Holocene is attested at several aceramic Neolithic sites, notably Parekklisha *Shillourokambos*, Khirokhitia *Vouni*, and Kritou Marottou *Ais Giorkis*. These early settlers would have encountered few endemic wild animals. Late Pleistocene fauna included pygmy elephants, dwarf hippos, genets, and shrews [1, 2], though the elephants and hippos had been extirpated, possibly by humans [3] prior to Neolithic colonization. Cyprus stands as an intriguing example of the dispersal of key taxa from the Near East into Europe during the first half of the Holocene, as humans transported them from the mainland, perhaps through multiple introductions [4, 5]. In addition to domestic animals such as sheep (*Ovis aries*), goats (*Capra hircus*), cattle (*Bos taurus*), pigs (*Sus scrofa*)—and even cats (*Felis silvestris lybica*)—wild species including fallow deer (*Dama dama mesopotamica*) and fox (*Vulpes vulpes*) also were imported [2, 5, 6].

Questions persist surrounding the domestic status and management of these taxa from the time of their introduction in the Neolithic (c. 9,000 cal BCE), into later periods, including the Bronze Age (c. 2,500 cal BCE). Were wild goats and pigs introduced to establish stock, and then hunted? Or, if they were indeed tame, how intensively were they husbanded? Were fallow deer populations managed alongside other animals, and if so, to what extent? For instance, Vigne and others used demographic profiles of faunal remains at *Shillourokambos* (8750–6830 cal BCE) to determine that sheep and cattle were present in their domestic forms and were managed as such, while all of the goats and about half of the pigs at the site were wild or feral [2]. At *Ais Giorkis* (7950–7060 cal BCE), fallow deer are the dominant species in the faunal assemblage, and Simmons [3] suggests hunting of deer followed by pig husbandry as the main modes of animal food production, with some herding of sheep and goat. Simmons reasons that the low proportion of cattle bones may reflect their ritual use. Though smaller body size generally is considered indicative of domestic status, it is also a hallmark of island populations. Therefore, the management of any species must consider multiple lines of evidence. Demographic profiles paired with stable isotope data provide further insight into changing management practices and the utilization of domestic, feral, and wild animals on the island throughout the duration of the Holocene.

The Early and Middle Bronze Ages of Cyprus (cf. Early and Middle Cypriot, c. 2500–1500 BCE) were characterized by dispersed villages practicing agriculture and copper metallurgy [7]. The best excavated settlements from these periods include Alambra *Mouttes* [8, 9], Marki *Alonia* [10, 11], Sotira *Kaminoudhia* [12], and Politiko-*Troullia* [13]. These villages were likely politically autonomous, yet linked economically, prior to the development of urbanism on Cyprus in the Late Bronze Age/Late Cypriot, c. 1500–1050 BCE [7]. At the Middle Bronze Age site of Politiko-*Troullia*, inhabitants utilized a range of both wild and domestic resources by practicing agriculture and arboriculture, harvesting fuelwood, hunting wild deer and feral pig, and herding sheep, goat, and cattle [13–16]. Spatial patterns of bone deposition at *Troullia* indicate communal deer feasting in public courtyards, with routine consumption of sheep, goat, and cattle in domestic contexts [17].

On some Mediterranean islands, particularly in the Aegean, many Neolithic economies depended on imported domestic resources, before incorporating wild taxa in greater numbers later in time [18–20]. Since sheep, goat, cattle, and pig represent introduced domesticates at *Troullia*, how intensively were these species managed by the local population? If feasting focused on deer, were these herds also managed, or did they represent the spoils of the hunt? Stable isotope analysis of δ^13^C and δ^15^N from both animal and plant remains has the potential to address these questions by providing data for diet and therefore related herd management strategies. Here, we present one of the first and most comprehensive studies to generate stable isotope data for archaeological fauna and modern and archaeological plant material on Cyprus.

## Materials and methods

### Field methods and faunal sampling

**Fig 1:**
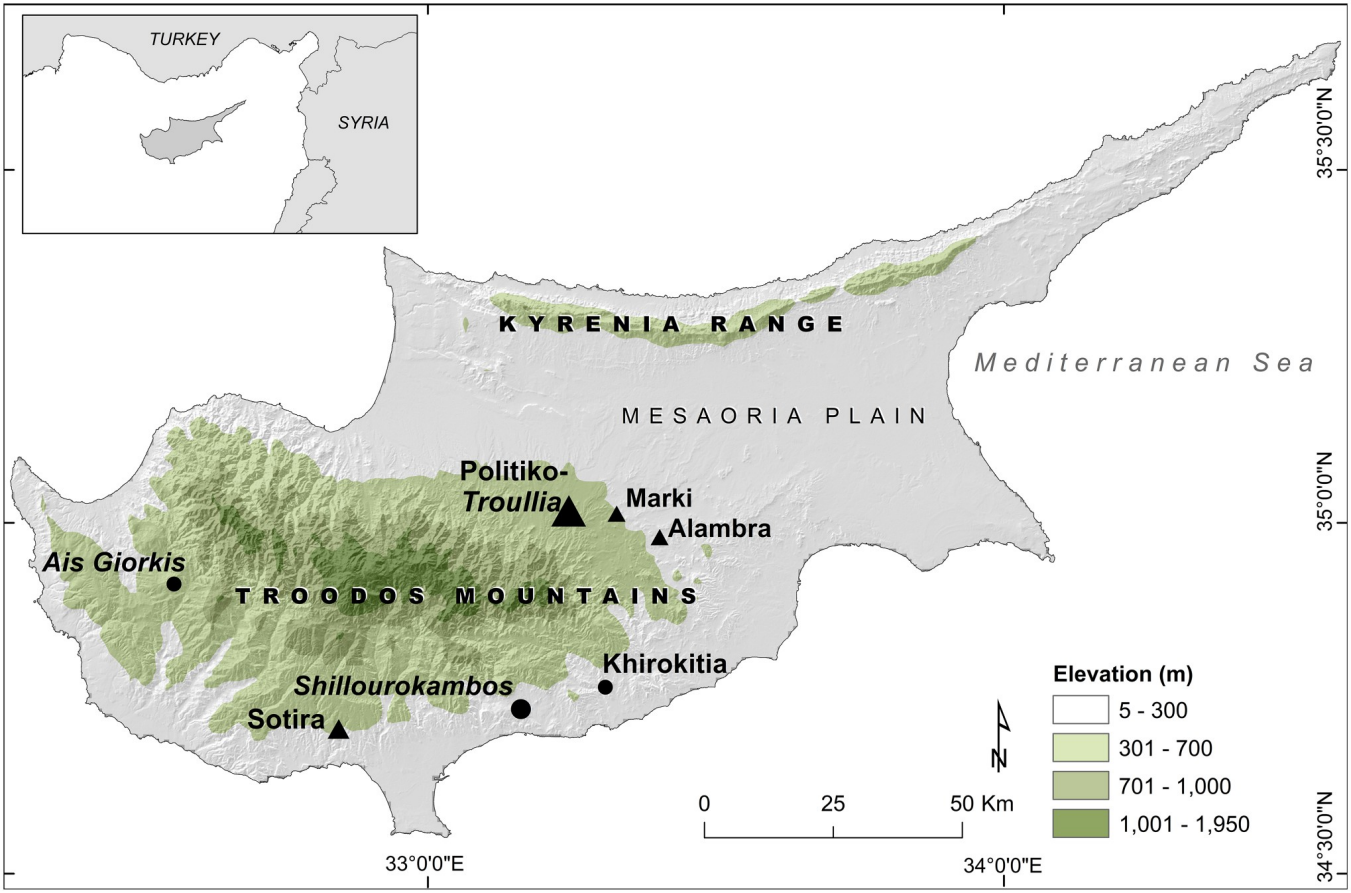
Map of Cyprus showing locations of Bronze Age sites (triangles), including Politiko-*Troullia*, and Neolithic sites (circles) mentioned in text. This figure was produced using ESRI ArcGIS version 10.8.x.

The main village component of Politiko-*Troullia* covers approximately 2 ha on an alluvial terrace in the foothills of the Troodos Mountains of central Cyprus (Fig 1). Bronze Age material culture associated with the site is spread over 20 ha on extensively terraced hillsides that suggest intensive pre-modern cultivation of the local landscape [21–24]. The village architecture of *Troullia* West is comprised of six stratified phases of construction and reconstruction in sediments more than three meters deep. Bayesian modeling of 25 calibrated AMS ages places these six contiguous phases of occupation at *Troullia* between about 2050 and 1850 cal BCE [13, 25]. All excavated sediments were dry-sieved through 0.5 cm wire mesh to ensure maximum recovery of material evidence from all six phases. Carbonized seeds and charcoal were recovered from shallow localized surfaces or features with visible burned organic content using non-mechanized water flotation. In this paper, we evaluate the role of cultivated crops and the surrounding landscape in animal husbandry practices by using stable isotope values to infer herding strategies and domestic status.

The faunal remains excavated from Politiko-*Troullia* were identified and sampled in our field lab in Pera Orinis, Cyprus. Whenever possible, we chose unarticulated same-sided bone elements within species to avoid sampling the same individual more than once. Multiple skeletal elements from the same context and taxon were analyzed only when we could differentiate individuals confidently based on qualitative characteristics of the samples. Analyzed taxa include Mesopotamian fallow deer (*Dama dama mesopotamica*), sheep (*Ovis aries*), goat (*Capra hircus*), cattle (*Bos taurus*), pig (*Sus scrofa*), fox (*Vulpes vulpes*), and owl, most likely barn owl (*Tyto alba*) (one sample). A total of 217 bones from stratigraphically secure contexts were sampled for stable isotope analysis. All necessary permits were obtained from the Department of Antiquities, Republic of Cyprus for the described study, which complied with all relevant regulations.

### Bone collagen

Collagen extraction was performed at the University of Georgia (UGA) Quaternary Isotope Paleoecology (QUIP) Lab using a modified Longin method by demineralizing fragmented bone weighing approximately 0.5 g in 0.5M HCl for several days [26]. The acid was changed regularly until the sample floated and was soft. Samples were rinsed using Type I water and gelatinized in pH 3.0 water at 75°C for 48 hours. Each sample was filtered using an EZEE filter and the supernatant liquor was freeze dried. Subsamples of freeze-dried collagen were weighed in tin capsules at the Center for Applied Isotope Studies (CAIS), UGA and analyzed on a Costech Elemental Analyzer coupled to a Finnigan Delta IV Plus IRMS. Stable carbon is reported relative to the standard Vienna Pee-Dee Belemnite (VPDB), and stable nitrogen is reported relative to ambient atmospheric N_2_ (AIR). Standards were supplied from the National Institute of Standards & Technology (NIST). Polyethylene foil (δ^13^C = -32.2‰) and sucrose (δ^13^C = -10.5‰) were used as standards for carbon, and ammonium sulfate (δ^15^N = 20.4‰) and potassium nitrate (δ^15^N = 4.7‰) were used for nitrogen. Two internal standards (spinach: δ^15^N = -0.3±0.2‰; δ^13^C = -27.2±0.1‰, and protein: δ^15^N = 8.0±0.1‰; δ^13^C = -17.6±0.03‰) were also used. Long-term precision of the IRMS is ≤0.2‰ for δ^15^N and ≤0.1‰ for δ^13^C. Of the 217 bones analyzed, 130 produced useable data for a success rate of 60%. In the case of failed samples, poor collagen preservation led to low collagen yield, low amounts of %C or %N, or C:N ratios outside the acceptable range of 2.9–3.6‰ (cf. [27]). Successful samples produced stable isotope data for the following skeletal elements: tibia (n = 37), humeri (n = 67), radii (n = 9), femora (n = 2), astragali (n = 12), a tibiotarsus (n = 1), a calcaneus (n = 1) and a petrous process (n = 1)(S1 Table).

### Modern and ancient plants

Modern *Triticum* and *Hordeum* seeds were sampled from non-irrigated, non-manured fields within 6 km of Politiko-*Troullia* in the summers of 2017, 2018, and 2019; collection points were georeferenced for spatial analysis. Samples were dried, freeze-dried, and then ground with a mortar and pestle at either the University of Georgia or University of North Carolina Charlotte (UNCC) laboratories. Samples were analyzed for total C, total N, δ^13^C, and δ^15^N at CAIS, UGA on a Costech Elemental Analyzer coupled to a Finnigan Delta IV Plus IRMS. Stable carbon is reported relative to VPDB and stable nitrogen is reported relative to AIR based on the NIST standards listed above. The modern plant stable isotope data presented below have been corrected for the Suess effect by +2‰ in δ^13^C (cf. [28]).

Additional stable isotope values were derived for 16 carbonized seed samples from Politiko-*Troullia* that were analyzed for ^14^C AMS ages. These include 11 samples analyzed at the University of Arizona Accelerator Mass Spectrometry Laboratory (δ^13^C only), and five samples analyzed at the University of Georgia Center for Applied Isotope Studies (δ^13^C and δ^15^N). To minimize the potential for chronological mixing, samples were selected as often as possible from relatively shallow localized deposits in or on burned surfaces. All recovered plant remains 0.25 mm or larger were sorted under a binocular microscope at 6 to 40x magnification, and identified using Fall’s personal reference collection, as well as comparison with published literature following established methods of floral recovery and analysis [14, 15, 29–31]. At the University of Arizona, δ^13^C was measured with an isotope ratio mass spectrometer. At the University of Georgia, these values were determined using a Delta-V elemental analyzer isotope ratio mass spectrometer (EA-IRMS). The archaeological plant stable isotope data presented below have been corrected using a charring offset of -0.11‰ for δ^13^C [32](S2 Table).

## Results

### Bone collagen at Politiko-*Troullia*

**Fig 2:**
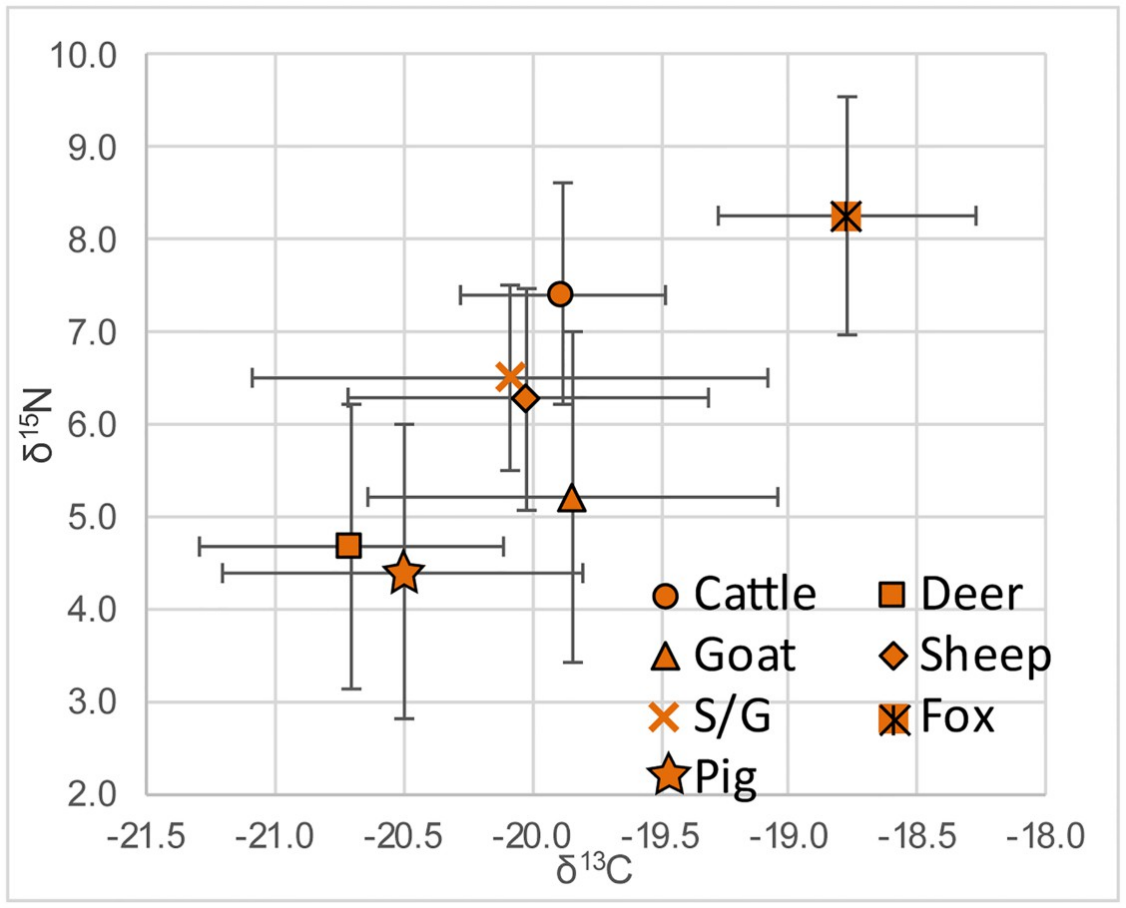
Means and standard deviations for δ^13^C and δ^15^N from bone collagen according to taxa at Politiko-*Troullia*, Cyprus. Bone collagen samples n = 140; deer (54), cattle (8), sheep (31), goat (27), sheep/goat (8), pig (8), fox (4).

The average δ^13^C value across all taxa is -20.2±0.8‰ with a maximum of -18.1‰ and a minimum of -22.3‰. The average δ^15^N is 5.7±1.9‰ with a maximum of 13.5‰ and a minimum of 1.8‰. Mean, minimum, maximum, and standard deviation values are listed in Table 1, and means and standard deviations for each taxonomic group are presented in Fig 2. There does not appear to be any relationship between isotopic values and the skeletal element sampled; variability is therefore attributed to individual diet and surrounding environment. With the exception of the single owl specimen, the data for both δ^13^C and δ^15^N are normally distributed for all taxa (Shapiro-Wilk) as well as for within-taxa groupings. We use Tukey’s HSD to identify significant differences in means between taxonomic groups, but exclude the sheep/goat category since it includes samples with combined taxa.

**Table 1:** Mean, minimum, maximum, and standard deviation values of δ^13^C and δ^15^N from bone collagen for animal taxonomic groups. A single value for an owl bone is excluded (δ^13^C = -18.4‰, δ^15^N = 13.5‰).

	Deer (n = 43)		Cattle (n = 8)		Goat (n = 27)		Sheep (n = 31)		Sheep/Goat (n = 8)		Pig (n = 8)		Fox (n = 4)	
	δ13C	δ15N	δ13C	δ15N	δ13C	δ15N	δ13C	δ15N	δ13C	δ15N	δ13C	δ15N	δ13C	δ15N
**Mean**	-20.8	4.5	-19.9	7.4	-19.8	5.2	-20.0	6.3	-20.1	6.5	-20.5	4.4	-18.8	8.3
**Min**	-22.3	2.1	-20.8	6.0	-22.0	1.8	-21.4	3.4	-21.3	5.0	-21.6	2.7	-19.2	6.9
**Max**	-19.6	8.9	-19.2	9.4	-18.1	8.5	-18.4	8.3	-18.1	8.4	-19.7	6.4	-18.2	9.9
**SD**	0.6	1.5	0.4	1.2	0.8	1.8	0.7	1.2	1.0	1.0	0.7	1.6	0.5	1.3

#### Bone collagen: δ^13^C

**Fig 3:**
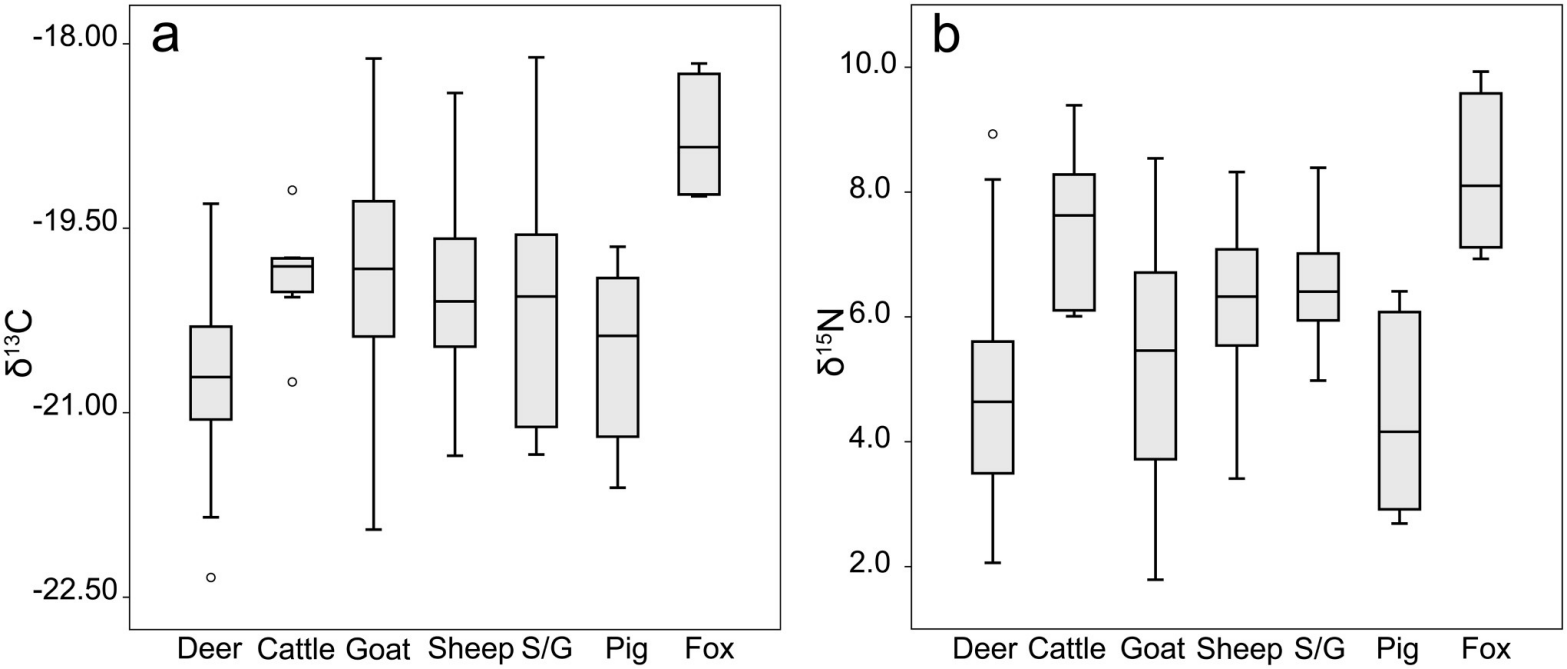
(a) Boxplots of bone collagen δ^13^C medians and quartiles for each taxon at Politiko-*Troullia*, Cyprus (circles indicate outliers). Owl not shown. (b) Boxplots of bone collagen δ^15^N medians and quartiles for each taxon at Politiko-*Troullia*, Cyprus. Owl not shown.

Mean values of δ^13^C for the fauna from Politiko-*Troullia*, which are typical for herbivores consuming a majority of C3 plant material, fall between -19.8‰ (goat) and -20.8‰ (fallow deer) (Table 1). Notably, the single raptor analyzed, as well as the four foxes, have more positive δ^13^C values (approximately +1‰), as expected for carnivores due to their higher trophic level. The domestic herbivores (cattle, sheep, goat, and mixed sheep/goat) show similar distributions of values, with largely overlapping ranges (Fig 3a), though cattle show the least variability. The mid-range of values (i.e., the middle two quartiles) for mixed sheep/goat resembles that of sheep more closely than goat, suggesting that sheep constitute the majority in this category. Pigs have a slightly more negative mean δ^13^C value (-20.5‰) than the means for cattle, sheep, goats, or sheep/goat, though there is significant overlap of the ranges for individual taxa. The mean δ^13^C of pigs is not significantly different from the means for other domestic taxa. Pigs also have the smallest overall range in δ^13^C with the exception of foxes. Finally, deer have the most negative δ^13^C values and the least amount of overlap with other taxa. The mid-range of deer values is distinct from those of sheep, goats and cattle, and overlaps only the lower range of sheep/goat and pig (Fig 3a). The mean δ^13^C value for deer is significantly different from those of all other taxonomic groups, with the exception of pig (Tukey’s HSD, p ≤0.01).

#### Bone collagen: δ^15^N

The carnivores have more positive δ^15^N values than all of the herbivore categories due to their higher trophic level (+2–3‰ enrichment per step), with a single owl at 13.5‰ and a mean value for foxes of 8.3‰. The means for cattle (7.4‰), sheep/goat and sheep (6.5‰ and 6.3‰), and goat (5.2‰) descend in increments of about 1‰ δ^15^N (Fig 3b). The mid-range distributions for all herbivores, except cattle, are distinct from the mid-range of foxes. As seen also in the δ^13^C data, distributions of δ^15^N values for sheep and sheep/goat suggest that the sheep/goat category includes more sheep than goat. Deer (4.5‰) and pig (4.4‰) have the lowest means and mid-range distributions. The elevated mean δ^15^N for cattle is significantly different than the means for the other herbivores (Tukey’s HSD p≤0.01), with the exception of sheep, where the difference is less pronounced (Tukey’s HSD, p = 0.5).

### Modern and ancient plants: δ^13^C and δ^15^N

**Fig 4:**
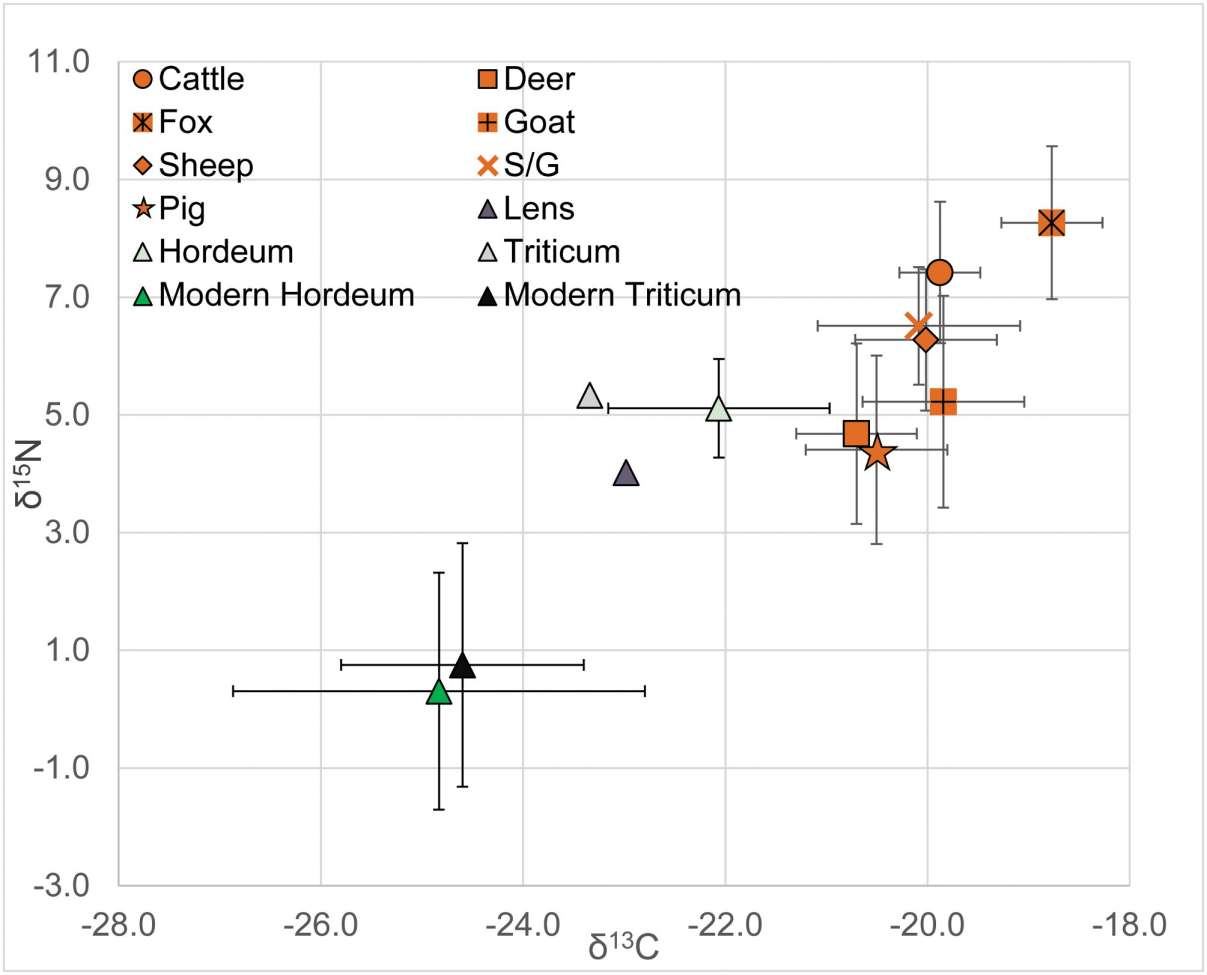
Means and standard deviations for δ^13^C and δ^15^N from animal bone collagen compared to means and standard deviations for δ^13^C and δ^15^N for archaeological seeds from Bronze Age Politiko-*Troullia*, Cyprus and modern cultivated Triticum and Hordeum collected in the vicinity of Politiko-*Troullia*, 2017–2019.

Stable isotope data for 21 modern *Triticum* and *Hordeum* seed samples from the vicinity of Politiko-*Troullia* produce δ^13^C and δ^15^N values within the expected ranges for C3 photosynthesis (mean δ^13^C = 24.7‰, sd = 1.6; mean δ^15^N = 0.6‰, sd = 2.0). Bronze Age seed samples from Politiko-*Troullia* (*n* = 4) provide a δ^13^C mean of -22.61‰ (sd = 0.9‰) and a δ^15^N mean of 4.9‰ (sd = 0.8‰). Results from modern cereals are compared to δ^13^C and δ^15^N values for the Politiko-*Troullia* seed samples in Fig 4.

## Discussion

Our study infers Bronze Age animal diet, herd management, and environmental conditions based on analysis of stable isotope data from animal bones and seed samples from Politiko-*Troullia*. In particular, we interpret values of δ^13^C and δ^15^N from domestic animals (sheep, goat, cattle, and pig) and wild deer. Plants absorb atmospheric CO_2_ such that stable carbon concentrations for modern and ancient plants can be useful indicators of relative water availability [33, 34]. The ratio of ^13^C to ^12^C, expressed as δ^13^C, serves as a proxy measure according to which lower values of δ^13^C are typically associated with increased water availability [35]. Herbivores consuming a predominantly C3 diet will have more negative δ^13^C values in their tissues, ranging from approximately -20 to -22‰ δ^13^C in bone collagen, in comparison to -14 to -10‰ δ^13^C for those consuming a predominantly C4 diet [36, 37]. Herbivore δ^13^C bone collagen values increase by an estimated +5‰ based on their diet [38] in comparison to omnivore and carnivore values, which are expected to be approximately +0–2‰ δ^13^C higher than those of their prey [39].

Nitrogen stable isotope values in non-N fixing plant foliage primarily reflect relative precipitation, temperature, and plant available N, with higher δ^15^N values associated with warmer, drier climatic conditions and higher soil δ^15^N [40]. In arid and Mediterranean environments, plant growth is generally limited according to the availability water and N, particularly in croplands [41]. Manuring is a common agricultural method that supplements or replaces organic N, and increases δ^15^N values [42]. Steeper slopes and other locations with undeveloped soils are expected to have lower N availability compared to locations with well-developed soils [42, 43]. The landscape surrounding Politiko-*Troullia* features steep slopes of Quaternary marine, alluvial, and Cretaceous limestone, with poorly developed soils and incised rills and drainages [44]. Extensive terracing is present along the slopes of Politko-*Koliokremmos*, providing increased area for agricultural production, and water and sediment retention adjacent to the relatively flat terrain of Politiko-*Troullia* [22]. Thus, faunal δ^15^N values are used primarily to infer animal diet and trophic level. However, elevated values can be caused by other factors such as increased aridity [45] or grazing on manured pastures or terraces [46–48]. Bones from terrestrial herbivores consuming only plant material tend to produce δ^15^N values of approximately 5–6‰. However, these values can range between 2‰ and 10‰ (e.g., [49]) and in some cases may vary more among individuals within a single taxonomic group than between animals at different trophic levels [50]. With these considerations in mind, an increase of 3–5‰ is expected with each step up in trophic level between prey and consumer [39, 51, 52].

### Diet and herd management

For the purpose of inferring animal diets and domestic status at Politiko-*Troullia*, it is useful to consider the possible offsets between stable isotope values from animal bones and those from ancient and modern vegetation. The average δ^13^C values for cattle (-19.9‰), goat (-19.8‰) and sheep (-20‰) all demonstrate an expected trophic offset up to +5‰ relative to domestic wheat and barley values derived from our sampling of both modern (-24.7‰) and ancient (-22.7‰) cultigens. These results compare well with δ^13^C values for 37 carbonized cereal and legume samples from Bronze Age Jordan, for example [29]. The δ^15^N offsets between values for archaeological cereal samples (5.2‰) and sheep, sheep/goat, and cattle bone collagen from Politiko-*Troullia* suggest the expected trophic increase of +2–3‰. In conjunction with the δ^13^C values from Politiko-*Troullia*, these results allude to a livestock diet based primarily on domestic crops. There is no offset between δ^15^N cereal values and those for goats (5.2‰), suggesting that their free-range diet may have consisted of a mixture of both domestic crops and wild forage. The lower mean δ^15^N values for deer (4.5‰) and pig (4.4‰) preclude the consumption of domestic plant taxa and suggest instead that these animals may have consumed only wild plants.

**Fig 5:**
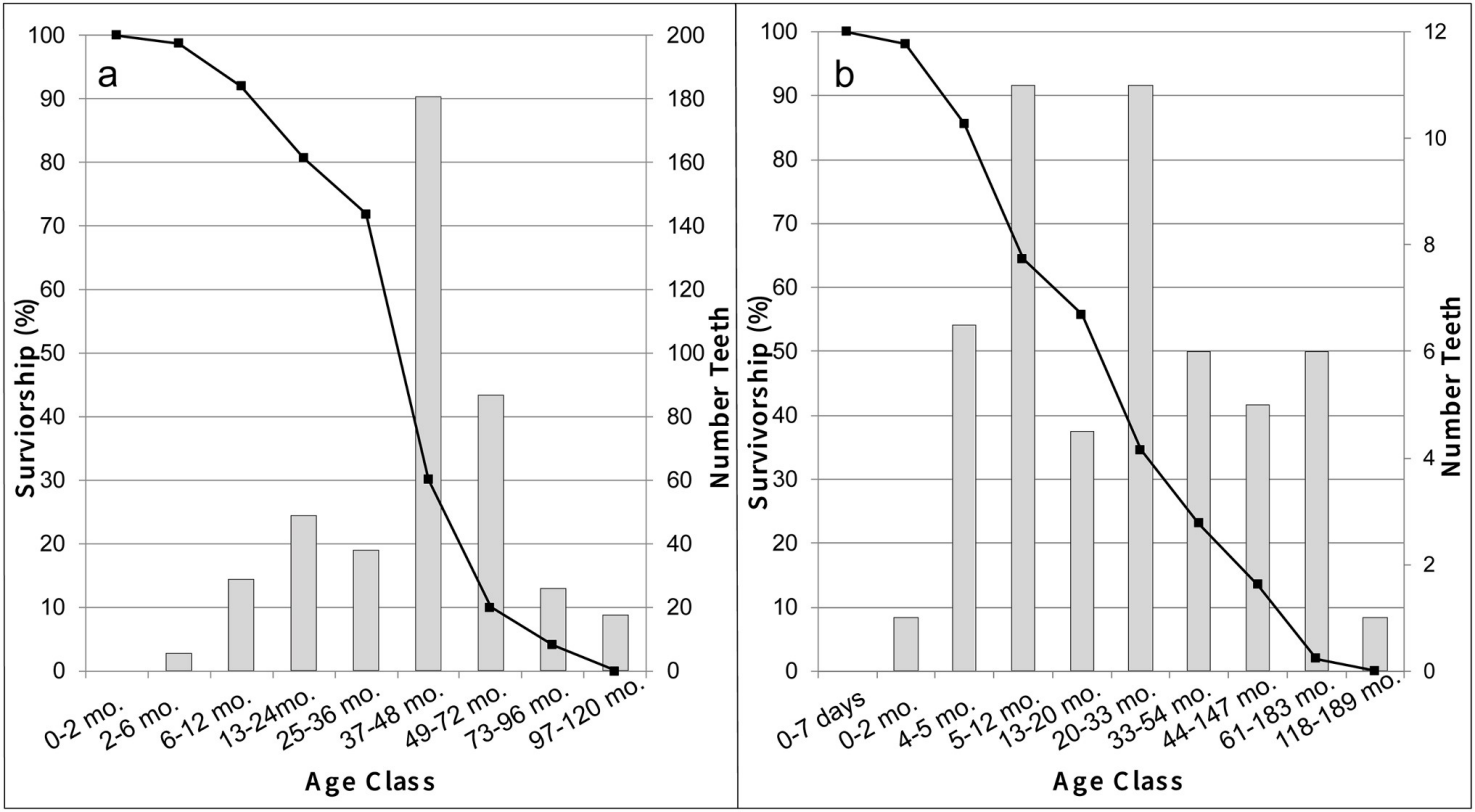
Survivorship curves (black lines) and tooth frequencies (gray bars) according to age classes for (a) sheep/goat and (b) fallow deer based on analysis of eruption and wear of teeth from Politiko-*Troullia*.

Demographic profiles were generated previously from analysis of *Ovis/Capra* and *Dama* teeth excavated at Politiko-*Troullia* [16]. Survivorship curves for these taxa are based on established criteria for tooth eruption and wear [53, 54]. The demographic data for *Ovis/Capra* show a pronounced spike in tooth frequency and a drop in survivorship among sheep and goats between three and six years of age (Fig 5a), both of which are consistent with culling of younger individuals as a major aspect of herd management. *Dama* teeth are distributed over a broader range of age classes, and survivorship declines more gradually between about 2 months and 5 years of age (Fig 5b). These patterns accord with mortality profiles associated with hunting of a wild animal population, which would have targeted larger bodied (i.e., older) animals that would have yielded greater amounts of meat.

#### Goats, sheep, and cattle

Amongst the Bronze Age mainstay domesticates (goats, sheep, and cattle), goats exhibit the largest range in δ^13^C values, followed by sheep. This pattern accords with the known dietary flexibility of goats and the well-attested practice of allowing domesticated goats and (to a lesser extent) sheep, to roam freely across pasturelands (e.g., [55]). In contrast, the more constrained δ^13^C range of cattle may indicate that these animals were penned and therefore accessed more limited vegetation sources and/or were provisioned with hay, an argument supported by the δ^15^N data (discussed below). Survivorship curves for these taxa at Politiko-*Troullia* affirm their domestic status (cf., [16]), though each species was managed differently. The striking spread of δ^15^N values across multiple herbivorous taxa may reflect a combination of wild and domesticated individuals, as well as mixed methods of husbandry for the domesticates. The elevated δ^15^N mean for cattle supports the scenario proposed above of limited grazing space and/or provisioning of cattle with feed, as their grazing may have been constrained in an area that was self-fertilized. Alternatively, they may have been provided hay from manured fields. Either possibility would lead to increased δ^15^N values. Sheep and the mixed sheep/goat category have the next highest δ^15^N values for herbivores. If the majority of individuals in the mixed category were sheep, this result suggests that sheep may have fed in the same areas as cattle, but their movements were not as constrained. Goats have the widest range in δ^15^N values, reinforcing inferences of flexible foraging and high freedom of movement for this species.

#### Fallow deer

Fallow deer and pig have essentially the same low mean for δ^15^N, suggesting that deer and pigs were foraging in more wooded habitats, and were not provisioned by humans and did not graze on manured land. Fallow deer δ^13^C values at Politiko-*Troullia* are significantly more negative than those of other taxa, suggesting they were consuming vegetation from a closed, wooded environment. This pattern accords with the inference based on survivorship curves that the villagers at *Troullia* hunted wild deer in the surrounding countryside, rather than managing a husbanded population, as has been reported for this species at other sites and time periods across the Near East and Europe (see [56] for a discussion of the osteometric evidence). Osteometric analysis of fallow deer from a variety of sites on Cyprus shows that they diminished in size through time following their importation to Cyprus in the early Neolithic [56, 57]. The zooarchaeological record for fallow deer (cf. Deer Bone Database, Zooarchaeology at Nottingham, https://www.nottingham.ac.uk/zooarchaeology/deer_bone/) shows a post-Neolithic gap in deer remains on Cyprus followed by osteometric data for larger animals in the Bronze Age, possibly suggesting the introduction of a new deer population. Though the fallow deer at Politiko-*Troullia* are relatively larger than would be expected for an endemic population (similar in size to those found at Pre-Pottery Neolithic B *Shillourokambos*; see [16]), there is no support for the argument that the deer at Politiko-*Troullia* themselves were imported since their isotopic signature appears “local” based on comparison with values for modern and ancient vegetation. The *Troullia* deer values agree with patterns of geographic variation reported by previous studies, in which fallow deer in northern and western Europe had more negative δ^13^C (below -22‰) and higher δ^15^N values (5–8‰) than in Turkey, which had average values of over -20.5‰ δ^13^C and 4.5‰ δ^15^N [58–60].

#### Pigs

The δ^13^C data suggest that pig exploitation at Politiko-*Troullia* included both domestic and feral individuals, who may have consumed some forage similar to that eaten by domesticates, as well as woodland vegetation. This conclusion is bolstered by osteometric and long bone fusion evidence of large body sizes and a predominance of older adult individuals in the assemblage [16]. Thus, the evidence for swine suggests a dual strategy of hunting as well as husbandry at Politiko-*Troullia*. Additionally, the lower δ^15^N signal in pigs is notable given their omnivorous diet, which should produce elevated δ^15^N relative to the values for herbivorous taxa (cf. [61]). This may suggest consumption of a leguminous diet [62] in addition to foraging in forested areas with poorly developed soils. A possible parallel is reported for Neolithic Çatalhöyük, where Pearson et al. [63] found that pigs had the lowest δ^15^N compared to all other medium-sized mammals at the site, and postulate that the main contributor of protein to their diet may have been roots and rhizomes, which also have lower relative δ^15^N values.

#### Plants and animal bone collagen summary

When compared with stable isotope data for modern and archaeological vegetation, the isotope signatures for archaeological fauna at Politiko-*Troullia* demonstrate herbivorous, C3-based diets. The average δ^13^C values for cattle, goat, and sheep suggest that domestic livestock consumed a diet with a significant proportion of crop taxa (e.g., as hay), in addition to grazing on grass and other forage. The more negative δ^13^C values for deer and pig suggest a diet based primarily on wild plants not supplemented by domestic crop species. There is a notable offset of +2–3‰ between the δ^15^N values for archaeological seeds as compared to modern domestic taxa. Enrichment of ^15^N can be caused by the practice of manuring in contrast to the use of modern inorganic fertilizers [64]. The higher δ^15^N values for the cattle and sheep support an argument for a practice of more intensive management, including the possibilities of grazing on manured fields or feeding of penned animals. This stands in contrast to the lower values for goats, which likely reflect a free-ranging lifestyle that incorporated both domestic feed and wild vegetation.

### Politiko-*Troullia* in context: Comparison to other stable isotope data from Cyprus

In order to contextualize the stable isotope data from Bronze Age Politiko-*Troullia*, we compare our results with data from other isotopic studies performed on faunal remains excavated on Cyprus from Neolithic Kritou Marottou *Ais Giorkis* (hereafter *Ais Giorkis*), which dates to approximately 9900 BP [65], and Early Bronze Age Marki *Alonia* dating to c. 2400–1900 BCE [66]. Marki *Alonia* is close to Politiko-*Troullia* in both a geographic and temporal sense, lying just 8km to the east (cf. Fig 1), with occupation beginning before and overlapping with that of Politiko-*Troullia*. *Ais Giorkis*, located in the southwestern foothills of the Troodos mountains, is much earlier in age. We note two other modest examples of stable isotope data from Cyprus, which we do not include for comparison with Politiko-*Troullia*. The first pertains to animal bones from Neolithic and Chalcolithic Limassol (n = 3) that were not identified to taxonomic level beyond “herbivore” [67]. The second is from a review of stable isotope analyses of humans and fauna in [68], which includes mean faunal values from Neolithic Khirokitia (n = 4) for δ^13^C = -21.0±1.5‰ and δ^15^N = +7.0±0.8‰, which were estimated from a figure in [69].

**Fig 6:**
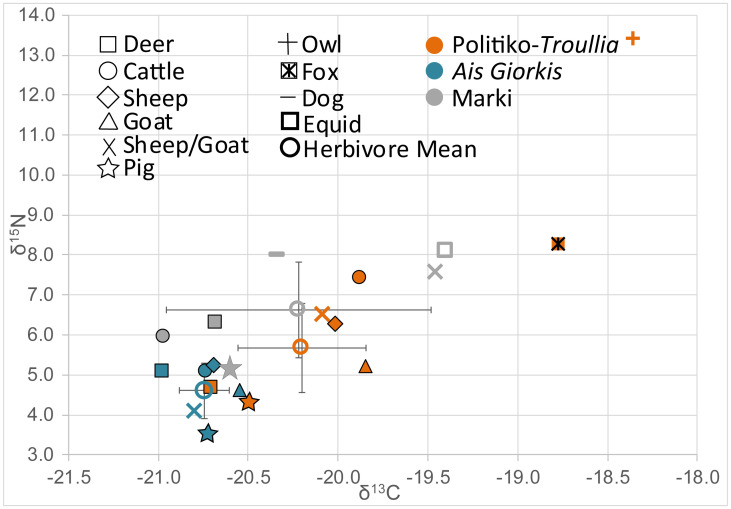
Means for δ^13^C and δ^15^N from bone collagen according to taxon, plus standard deviations for herbivores. Politiko-*Troullia* (PT), *Ais Giorkis* (AG) (data from [65]) and Marki *Alonia* (MA) (data from [66]).

Overall, the mean stable isotope values for herbivorous fauna at Politiko-*Troullia*, Marki *Alonia*, and *Ais Giorkis* fall within the expected ranges for animals consuming C3 diets (Table 2, Fig 6). The mean δ^13^C values for herbivores at Politiko-*Troullia* and Marki *Alonia* are the same (-20.2‰), while that for *Ais Giorkis* herbivores is only slightly more negative (-20.7‰). Stable nitrogen ratios are noticeably more varied, with *Ais Giorkis* having the lowest mean at +4.6‰ δ^15^N. Fauna at Politiko-*Troullia* are higher at +5.7‰ δ^15^N, followed by Marki *Alonia* at +6.6‰ δ^15^N. At *Ais Giorkis*, mean values vary less between taxa than at the other sites, perhaps suggesting fewer differences in management between animal species at this village.

**Table 2:** Mean and standard deviation of herbivore taxa at three Cypriot sites.

Site	N Herbivore Samples	Mean δ13C	Mean δ15N
Politiko-*Troullia*	136	-20.2±0.4	5.7±1.1
Marki *Alonia*	18	-20.2±0.7	6.6±1.2
*Ais Giorkis*	70	-20.7±0.1	4.6±0.7

Comparison of these data according to taxon reveals several noteworthy patterns across all three sites. Deer (n = 21) have the most negative mean δ^13^C values, while their mean δ^15^N is similar to those of domestic cattle, sheep, and goat. In contrast, pigs (n = 14) are similar to cattle, sheep, and goat in δ^13^C, but notably more negative in δ^15^N. Carnivores from each site (fox, owl, and dog) all plot as more positive in δ^13^C and δ^15^N than the herbivores, as would be expected for their higher trophic level.

Though only modestly different, the more negative δ^13^C values at *Ais Giorkis* could be related to greater precipitation or its more wooded setting in comparison to the two other sites, or to wetter Neolithic climatic conditions. In turn, the overall elevated δ^15^N values at Politiko-*Troullia* and Marki *Alonia* could be due to a combination of factors that potentially included naturally more enriched soil nitrogen in their vicinities, local aridity, and/or the practice of penning/manuring. At Marki and *Troullia*, δ^13^C and δ^15^N values for both deer and pig are significantly more negative than those for sheep and goat. These results support an argument that deer were not managed and that feral pigs were common during the Bronze Age. Similarly, at Marki, while the pig mortality evidence is “characteristic of the management of domestic swine, feral pigs may well have existed in the area…and [may] have been hunted in an opportunistic way” [70: 274].

## Conclusions

The stable isotope data for animal bone collagen from Politiko-*Troullia* illustrates the varied strategies of animal exploitation and landscape utilization practiced by its agrarian population. When these results are integrated with other archaeological and zooarchaeological data, it appears that the residents of this Bronze Age village kept a small number of cattle regularly penned, which they provisioned with hay and water. Sheep may have been managed with the cattle, but also went out to pasture regularly, thereby incorporating other food into their diet. Goats likely had even more freedom to roam and forage over a wider range. Deer, which had previously been introduced to the island by humans, formed a hunted wild population in the woodlands surrounding the village and may have moved to other habitats during part of the year. Sheep and goat comprised the primary sources of meat at Politiko-*Troullia*. Cattle were used for dairying, traction, and meat, while managed and feral pigs provided a supplementary component of the human diet at *Troullia*. Meanwhile, targeted deer hunts supported communal feasting.

Our inferences of the dietary niches occupied by the animal taxa consumed at Politiko-*Troullia* are supported by quantitative analyses of the settlement’s faunal assemblages and stable isotopic analyses of animal bone collagen in conjunction with stable isotope data for modern and ancient cultivated plant taxa. This study provides the most robust set of stable isotope data for Bronze Age animal management on Cyprus. Our analyses illuminate agrarian practices and landscapes during the centuries prior to the emergence of Cyprus’s first cities and urbanized society. In these capacities, our analysis of animal and plant remains from Politiko-*Troullia* illustrates the potential for stable isotopic inquiry to provide multi-faceted archaeologically-based portraits of agrarian lifeways in ancient communities.

## Supporting information

S1 Table Bone collagen stable isotope data from Politiko-*Troullia*. (XLSX)

S2 Table Stable isotope data from modern and archaeological plants. (XLSX)

S1 Graphical abstract (TIF)

## References

[1] Hadjisterkotis E, Masala B. Vertebrate extinction in Mediterranean islets: an example from Cyprus. Biogeographia. 1996;18. doi: 10.21426/B618110414

[2] Vigne J-D, Carrère I, Briois F, Guilaine J. The Early Process of Mammal Domestication in the Near East: New Evidence from the Pre- Neolithic and Pre-Pottery Neolithic in Cyprus. Current Anthropology. 2011;52: S255–S271. doi: 10.1086/659306

[3] Simmons AH. Ais Giorkis: An unusual early Neolithic settlement in Cyprus. Journal of Field Archaeology. 2012;37: 86–103.

[4] Munro ND, Bar-Oz G, Meier JS, Sapir-Hen L, Stiner MC, Yeshurun R. The Emergence of Animal Management in the Southern Levant. Sci Rep. 2018;8: 9279. doi: 10.1038/s41598-018-27647-z 29915348 PMC6006362

[5] Zeder MA. Domestication and early agriculture in the Mediterranean Basin: Origins, diffusion, and impact. Proceedings of the National Academy of Sciences. 2008;105: 11597–11604. doi: 10.1073/pnas.0801317105 18697943 PMC2575338

[6] Vigne J-D, Carrere I, Saliege J-F, Person A, Bocherens H, Guilaine J, et al. Predomestic cattle, sheep, goat and pig during the late 9th and the 8th millennium cal. BC on Cyprus: preliminary results of Shillourokambos (Parekklisha, Limassol). In: Mashkour M, International Symposium on the Archaeozoology of Southwestern Asia and Adjacent Areas, editors. Archaeozoology of the Near East IV: proceedings of the fourth international symposium on the archaeozoology of southwestern Asia and adjacent areas. Groningen: Centre for Archaeological Research and Consultancy; 2000.

[7] Knapp AB. Prehistoric and Protohistoric Cyprus: Identity, Insularity, and Connectivity. Oxford: Oxford University Press; 2008.

[8] Coleman JE, Gale NH, Cornell University, editors. Alambra: a middle Bronze Age settlement in Cyprus: archaeological investigations by Cornell University 1974–1985. Jonsered: P. Aström; 1996.

[9] Lowe KM, Fogel AS, Sneddon A. Archaeological geophysical survey of a Prehistoric Bronze Age site in Cyprus (Alambra Mouttes)—applications and limitations. Archaeol Anthropol Sci. 2018;10: 1971–1989. doi: 10.1007/s12520-017-0508-3

[10] Frankel D, Webb JM, Adams R, Croft P, Simmons D, Smith MA, et al. Marki Alonia: An Early and Middle Bronze Age Town in Cyprus—Excavations 1990–1994. 1996.

[11] Frankel D, Webb JM. Marki Alonia: An Early and Middle Bronze Age Settlement in Cyprus: Excavations 1995–2000. Paul Åströms förlag; 2006.

[12] Swiny S, Rapp G, Herscher E, editors. Sotira Kaminoudhia: An Early Bronze Age Site in Cyprus. The American Schools of Oriental Research; 2003. doi: 10.5615/j.ctt2jc9wt

[13] Falconer SE, Fall PL. Household and community behavior at Bronze Age Politiko-Troullia, Cyprus. Journal of Field Archaeology. 2013;38: 101–119. doi: 10.1179/0093469013Z.00000000041

[14] Fall PL, Falconer SE, Klinge J. Bronze age fuel use and its implications for agrarian landscapes in the eastern Mediterranean. Journal of Archaeological Science: Reports. 2015;4: 182–191. doi: 10.1016/j.jasrep.2015.09.004

[15] Klinge J, Fall P. Archaeobotanical inference of Bronze Age land use and land cover in the eastern Mediterranean. Journal of Archaeological Science. 2010;37: 2622–2629. doi: 10.1016/j.jas.2010.05.022

[16] Metzger MC, Ridder E, Birch SEP, Falconer SE, Fall PL. Animal Exploitation and Community Behavior at a Middle Bronze Age Village on Cyprus. Archaeozoology of Southwest Asia and Adjacent Areas XIII Proceedings of the Thirteenth International Symposium, University of Cyprus, Nicosia, Cyprus, June 7–10, 2017. Lockwood Press; 2021. pp. 113–127. https://www.jstor.org/stable/j.ctv2d7x51d.11

[17] Falconer SE, Monahan EM, Fall PL. A Stone Plank Figure from Politiko-Troullia, Cyprus: Potential Implications for Inferring Bronze Age Communal Behavior. Bulletin of the American Schools of Oriental Research. 2014; 3–16. doi: 10.5615/bullamerschoorie.371.0003

[18] Leppard T, Pilaar Birch S. The insular ecology and palaeoenvironmental impacts of the domestic goat (Capra hircus) in Mediterranean Neolithization. Géoarchéologie des îles de la Méditerranée. 2016; 47–56.

[19] Pilaar Birch S. From the Aegean to the Adriatic: Exploring the Earliest Neolithic Island Fauna. The Journal of Island and Coastal Archaeology. 2017;13: 256–268. doi: 10.1080/15564894.2017.1310774

[20] Pilaar Birch SE, Atici L, Erdoğu B. Spread of domestic animals across Neolithic western Anatolia: New stable isotope evidence from Uğurlu Höyük, the island of Gökçeada, Turkey. PLOS ONE. 2019;14: e0222319. doi: 10.1371/journal.pone.0222319 31600208 PMC6786565

[21] Fall PL, Soto-Berelov M, Ridder E, Falconer SE. Toward a Grand Narrative of Bronze Age Vegetation Change and Social Dynamics in the Southern Levant. In: Levy TE, Jones IWN, editors. Cyber-Archaeology and Grand Narratives: Digital Technology and Deep-Time Perspectives on Culture Change in the Middle East. Cham: Springer International Publishing; 2018. pp. 91–110.

[22] Fall PL, Falconer SE, Galletti CS, Shirmang T, Ridder E, Klinge J. Long-term agrarian landscapes in the Troodos foothills, Cyprus. Journal of Archaeological Science. 2012;39: 2335–2347. doi: 10.1016/j.jas.2012.02.010

[23] Galletti CS, Ridder E, Falconer SE, Fall PL. Maxent modeling of ancient and modern agricultural terraces in the Troodos foothills, Cyprus. Applied Geography. 2013;39: 46–56. doi: 10.1016/j.apgeog.2012.11.020

[24] Ridder E, Galletti CS, Fall PL, Falconer SE. Economic and social activities on ancient Cypriot terraced landscapes. Journal of Environmental Management. 2017;202: 514–523. doi: 10.1016/j.jenvman.2016.12.037 28041874

[25] Falconer SE, Ridder E, Pilaar Birch SE, Fall PL. Prehistoric Bronze Age Radiocarbon Chronology at Politiko-Troullia, Cyprus. Radiocarbon. 2022. 10.1371/journal.pone.0275757 PMC9605021 36288284

[26] Longin R. New Method of Collagen Extraction for Radiocarbon Dating. Nature. 1971;230: 241. doi: 10.1038/230241a0 4926713

[27] Ambrose SH. Preparation and characterization of bone and tooth collagen for isotopic analysis. Journal of Archaeological Science. 1990;17: 431–451. doi: 10.1016/0305-4403(90)90007-R

[28] Dombrosky J. A ~1000-year 13C Suess correction model for the study of past ecosystems. The Holocene. 2020;30: 474–478. doi: 10.1177/0959683619887416

[29] Porson S, Falconer S, Pilaar Birch S, Ridder E, Fall P. Crop management and agricultural responses at Early Bronze IV Tell Abu en-Ni’aj, Jordan. Journal of Archaeological Science. 2021;133: 105435. doi: 10.1016/j.jas.2021.105435

[30] Fall PL, Falconer SE, Porson S. Archaeobotanical inference of intermittent settlement and agriculture at Middle Bronze Age Zahrat adh-Dhra’1, Jordan. Journal of Archaeological Science: Reports. 2019;26: 101884. doi: 10.1016/j.jasrep.2019.101884

[31] Falconer SE, Fall PL. Bronze Age Rural Ecology and Village Life at Tell el-Hayyat, Jordan. Oxford, England: British Archaeological Reports; 2006.

[32] Nitsch EK, Charles M, Bogaard A. Calculating a statistically robust δ13C and δ15N offset for charred cereal and pulse seeds. STAR: Science & Technology of Archaeological Research. 2015;1: 1–8. doi: 10.1179/2054892315Y.0000000001

[33] Farquhar GD, O’Leary MH, Berry JA. On the Relationship Between Carbon Isotope Discrimination and the Intercellular Carbon Dioxide Concentration in Leaves. Functional Plant Biol. 1982;9: 121–137. doi: 10.1071/pp9820121

[34] Ehleringer JR. 12—13C/12C Fractionation and Its Utility in Terrestrial Plant Studies. In: Coleman DC, Fry B, editors. Carbon Isotope Techniques. Academic Press; 1991. pp. 187–200.

[35] Wallace MP, Jones G, Charles M, Fraser R, Heaton THE, Bogaard A. Stable Carbon Isotope Evidence for Neolithic and Bronze Age Crop Water Management in the Eastern Mediterranean and Southwest Asia. PLOS ONE. 2015;10: e0127085. doi: 10.1371/journal.pone.0127085 26061494 PMC4464649

[36] Kellner CM, Schoeninger MJ. A simple carbon isotope model for reconstructing prehistoric human diet. American Journal of Physical Anthropology. 2007;133: 1112–1127. doi: 10.1002/ajpa.20618 17530667

[37] Lee-Thorp JA, Sealy JC, van der Merwe NJ. Stable carbon isotope ratio differences between bone collagen and bone apatite, and their relationship to diet. Journal of Archaeological Science. 1989;16: 585–599. doi: 10.1016/0305-4403(89)90024-1

[38] Koch PL, Fogel ML, Tuross N. Tracing the diets of fossil animals using stable isotopes. Lajtha K and Michener R H, eds Stable isotopes in ecology and environmental science. Blackwell; 1994. pp. 63–92.

[39] Bocherens H, Drucker D. Trophic level isotopic enrichment of carbon and nitrogen in bone collagen: case studies from recent and ancient terrestrial ecosystems. International Journal of Osteoarchaeology. 2003;13: 46–53. doi: 10.1002/oa.662

[40] Craine JM, Elmore AJ, Aidar MPM, Bustamante M, Dawson TE, Hobbie EA, et al. Global patterns of foliar nitrogen isotopes and their relationships with climate, mycorrhizal fungi, foliar nutrient concentrations, and nitrogen availability. New Phytologist. 2009;183: 980–992. doi: 10.1111/j.1469-8137.2009.02917.x 19563444

[41] Passioura JB. Review: Environmental biology and crop improvement. Functional Plant Biol. 2002;29: 537–546. doi: 10.1071/FP02020 32689499

[42] Amundson R, Austin AT, Schuur EAG, Yoo K, Matzek V, Kendall C, et al. Global patterns of the isotopic composition of soil and plant nitrogen: Global Soil and Plant N Isotopes. Global Biogeochem Cycles. 2003;17. doi: 10.1029/2002GB001903

[43] Vitousek PM, Shearer G, Kohl DH. Foliar 15N natural abundance in Hawaiian rainforest: patterns and possible mechanisms. Oecologia. 1989;78: 383–388. doi: 10.1007/BF00379113 28312585

[44] Fall PL, Falconer SE, Horowitz M, Hunt J, Metzger MC, Ryter D. Bronze Age Settlement and Landscape of Politiko-Troullia, 2005–2007. Report of the Department of Antiquities, Cyprus. 2008 pp. 183–208.

[45] Hartman G. Are elevated δ15N values in herbivores in hot and arid environments caused by diet or animal physiology? Functional Ecology. 2010;25: 122–131. doi: 10.1111/j.1365-2435.2010.01782.x

[46] Fraser RA, Bogaard A, Heaton T, Charles M, Jones G, Christensen BT, et al. Manuring and stable nitrogen isotope ratios in cereals and pulses: towards a new archaeobotanical approach to the inference of land use and dietary practices. Journal of Archaeological Science. 2011;38: 2790–2804. doi: 10.1016/j.jas.2011.06.024

[47] Fraser RA, Bogaard A, Schäfer M, Arbogast R, Heaton THE. Integrating botanical, faunal and human stable carbon and nitrogen isotope values to reconstruct land use and palaeodiet at LBK Vaihingen an der Enz, Baden-Württemberg. World Archaeology. 2013;45: 492–517. doi: 10.1080/00438243.2013.820649

[48] Kanstrup M, Thomsen IK, Mikkelsen PH, Christensen BT. Impact of charring on cereal grain characteristics: linking prehistoric manuring practice to δ15N signatures in archaeobotanical material. Journal of Archaeological Science. 2012;39: 2533–2540. doi: 10.1016/j.jas.2012.03.007

[49] Bocherens H. Isotopic tracking of large carnivore palaeoecology in the mammoth steppe. Quaternary Science Reviews. 2015;117: 42–71. doi: 10.1016/j.quascirev.2015.03.018

[50] Sponheimer M, Robinson T, Ayliffe L, Roeder B, Hammer J, Passey B, et al. Nitrogen isotopes in mammalian herbivores: hair δ15N values from a controlled feeding study. International Journal of Osteoarchaeology. 2003;13: 80–87. doi: 10.1002/oa.655

[51] Deniro MJ, Epstein S. Influence of diet on the distribution of nitrogen isotopes in animals. Geochimica et Cosmochimica Acta. 1981;45: 341–351. doi: 10.1016/0016-7037(81)90244-1

[52] Schoeninger MJ, DeNiro MJ. Nitrogen and carbon isotopic composition of bone collagen from marine and terrestrial animals. Geochimica et Cosmochimica Acta. 1984;48: 625–639. doi: 10.1016/0016-7037(84)90091-7

[53] Bowen F, Carden RF, Daujat J, Grouard S, Miller H, Perdikaris S, et al. Dama Dentition: A New Tooth Eruption and Wear Method for Assessing the Age of Fallow Deer (Dama dama). International Journal of Osteoarchaeology. 2016;26: 1089–1098. doi: 10.1002/oa.2523

[54] Payne S. Kill-off Patterns in Sheep and Goats: the Mandibles from Aşvan Kale. Anatol Stud. 1973;23: 281–303. doi: 10.2307/3642547

[55] Hadjikoumis A. Ethnoarchaeology as a Means of Improving Integration: An Ethnozooarchaeological Study from Cyprus and Its Contribution to the Integration of Zooarchaeology with Archaeobotany and Other Lines of Archaeological Evidence. In: Pişkin E, Marciniak A, Bartkowiak M, editors. Environmental Archaeology: Current Theoretical and Methodological Approaches. Cham: Springer International Publishing; 2018. pp. 181–198.

[56] Daujat J. Ungulate invasion on a mediterranean island: the cypriot mesopotamian fallow deer over the past 10 000 years. These de doctorat, Paris, Muséum national d’histoire naturelle. 2013. https://www.theses.fr/2013MNHN0015

[57] Croft PW. Game management in early prehistoric Cyprus. Zeitschrift fuer Jagdwissenschaft. 2002;48: 172–179. doi: 10.1007/BF02192406

[58] Madgwick R, Sykes N, Miller H, Symmons R, Morris J, Lamb A. Fallow deer (Dama dama dama) management in Roman South-East Britain. Archaeol Anthropol Sci. 2013;5: 111–122. doi: 10.1007/s12520-013-0120-0

[59] Sykes N, Ayton G, Bowen F, Baker K, Baker P, Carden RF, et al. Wild to domestic and back again: the dynamics of fallow deer management in medieval England (c. 11th-16th century AD). STAR: Science & Technology of Archaeological Research. 2016;2: 113–126. doi: 10.1080/20548923.2016.1208027

[60] Miller H, Carden RF, Evans J, Lamb A, Madgwick R, Osborne D, et al. Dead or alive? Investigating long-distance transport of live fallow deer and their body parts in antiquity. Environmental Archaeology. 2016;21: 246–259. doi: 10.1179/1749631414Y.0000000043

[61] Madgwick R, Mulville J, Stevens RE. Diversity in foddering strategy and herd management in late Bronze Age Britain: An isotopic investigation of pigs and other fauna from two midden sites. Environmental Archaeology. 2012;17: 126–140. doi: 10.1179/1461410312Z.00000000011

[62] Hamilton J, Thomas R. Pannage, Pulses and Pigs: Isotopic and Zooarchaeological Evidence for Changing Pig Management Practices in Later Medieval England. Medieval Archaeology. 2012;56: 234–259. doi: 10.1179/0076609712Z.0000000008

[63] Pearson JA, Bogaard A, Charles M, Hillson SW, Larsen CS, Russell N, et al. Stable carbon and nitrogen isotope analysis at Neolithic Çatalhöyük: evidence for human and animal diet and their relationship to households. Journal of Archaeological Science. 2015;57: 69–79. doi: 10.1016/j.jas.2015.01.007

[64] Choi W-J, Lee S-M, Ro H-M, Kim K-C, Yoo S-H. Natural 15N abundances of maize and soil amended with urea and composted pig manure. Plant and Soil. 2002;245: 223–232.

[65] DiBenedetto KE. Investigating Land Use by the Inhabitants of Western Cyprus During the Early Neolithic. Ph.D., University of Nevada, Las Vegas. 2018. https://www.proquest.com/docview/2124052325/abstract/AD07F4A594B040EEPQ/1

[66] Scirè-Calabrisotto C, Webb JM, Frankel D, Ricci P, Altieri S, Lubritto C. New evidence for diet and subsistence economy in Early and Middle Bronze Age Cyprus. Journal of Archaeological Science: Reports. 2020;33: 102518. doi: 10.1016/j.jasrep.2020.102518

[67] Voskos I, Vika E. Prehistoric human remains reviewed: Palaeopathology and palaeodiet in Neolithic and Chalcolithic Cyprus, Limassol district. Journal of Archaeological Science: Reports. 2020;29: 102128. doi: 10.1016/j.jasrep.2019.102128

[68] Goude G, Clarke J, Webb JM, Frankel D, Georgiou G, Herrscher E, et al. Exploring the potential of human bone and teeth collagen from Prehistoric Cyprus for isotopic analysis. Journal of Archaeological Science: Reports. 2018;22: 115–122. doi: 10.1016/j.jasrep.2018.09.018

[69] Lange-Badré B, Le Mort F. Isotopes stables du carbone et de l’azote et éléments traces indicateurs du régime alimentaire de la population néolithique de Khirokitia (Chypre). L’homme préhistorique et la mer: actes du 120e Congrès national des sociétés historiques et scientifiques section de pré- et protohistoire, Aix-en Provence (23–26 Octobre 1995). Paris: C.T.H.S; 1998. pp. 415–426.

[70] Croft, P. Animal Bones. In: Frankel D, Webb JM, editors. Marki Alonia: An Early and Middle Bronze Age Settlement in Cyprus: Excavations 1995–2000. Paul Åströms förlag; 2006. pp. 263–281.

