# Supplementary figures and images for: Herd management and subsistence practices as inferred from isotopic analysis of animals and plants at Bronze Age Politiko-Troullia, Cyprus
Source: PLoS One. 2022 Oct 26;17(10):e0275757. doi: 10.1371/journal.pone.0275757 (PMC9605021; doi:10.1371/journal.pone.0275757)

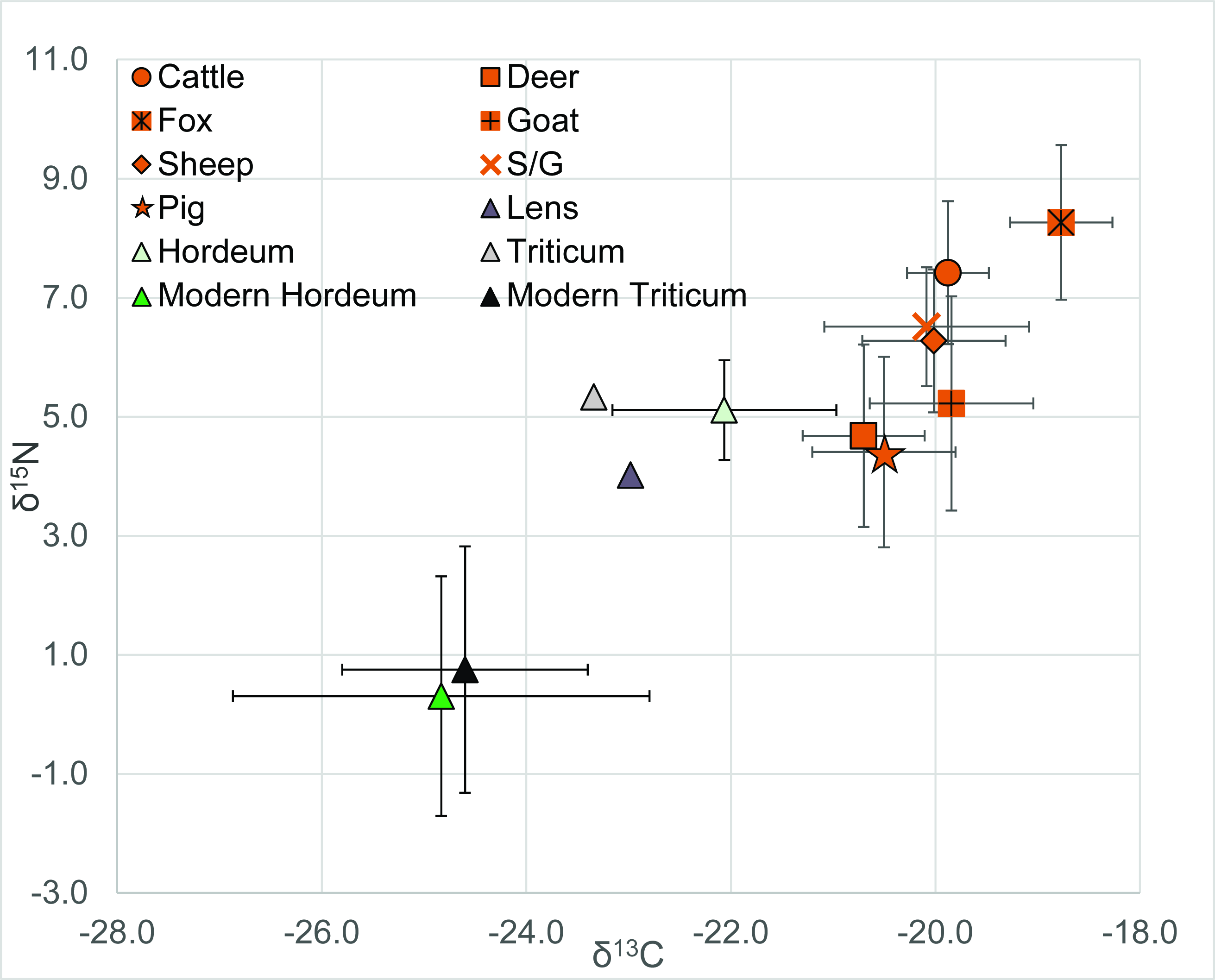

Supplement: S1 Graphical abstract — (TIF) [file pone.0275757.s003.tif]
